# Sustainable COVID-19 Mitigation: Wuhan Lockdowns, Health Inequities, and Patient Evacuation

**DOI:** 10.34172/ijhpm.2020.63

**Published:** 2020-04-28

**Authors:** Lee Liu

**Affiliations:** ^1^School of Geoscience, Physics and Safety, College of Health, Science and Technology, University of Central Missouri, Warrensburg, MO, USA.; ^2^School of Environment, Northeast Normal University, Changchun, Jilin, China.

**Keywords:** COVID-19, Lockdown, Pandemic, Health Inequity, China

## Abstract

The world is urgently looking for ways to flatten the coronavirus disease 2019 (COVID-19) curve, and many governments have resorted to implementing strict lockdowns, as researchers show the effectiveness of China’s approaches in containing the virus. However, this paper argues that the draconian lockdowns instituted in Wuhan, Hubei, China, may have actually contributed to intensifying patient surges and incapacitating local health systems. Medical aids were rushed to Hubei and new hospitals were rapidly built, however, the healthcare system was still unable to match the staggering increase of patients in the early stages of the lockdowns. The paper proposes using patient evacuation to enhance sustainable COVID-19 mitigation during lockdowns. It demonstrates that patients in Hubei could have been transported to other Chinese provinces where hospitals were under-utilized. This could have theoretically saved thousands of lives by reducing inequities between Hubei and the rest of China in healthcare capacity for treating COVID-19 patients.

## Introduction

China’s effective control of coronavirus disease 2019 (COVID-19) has largely been attributed to the draconian lockdowns and travel restrictions instituted nationwide.^1-3^ Amid increasing large-scale lockdowns around the world, current literature debates whether other countries should, or will even be able to, follow China’s example to flatten the coronavirus curve.^3,4^ China’s lockdowns started in Wuhan on January 23, 2020, and quickly expanded to the entire Hubei province and other provinces. By April 8, 2020, 78 days later, Wuhan lockdowns were mostly lifted, weeks after the coronavirus had been largely brought under control. It is time to look back and draw lessons from the Chinese experience that may benefit current and future pandemic control. While many have examined China’s COVID-19 mitigation, none have done so from a sustainability perspective. This paper explores sustainable COVID-19 mitigation with attention to health inequities associated with lockdowns in Wuhan and Hubei and the possible benefits of patient transport, in the context of the United Nations (UN) 2030 Agenda.^5^

## Methods

The UN 2030 Agenda provides guidance for countries to achieve Sustainable Development Goals (SDGs) by 2030.^5^ Interconnected and interdependent, many of the 17 SDGs are directly or indirectly related to health policy and management. In particular, SDG 3 is to “ensure healthy lives and promote well-being for all.” Accordingly, this paper defines sustainable COVID-19 mitigation as measures that ensure healthy lives and promote well-being for everyone and contribute to the development of sustainable, inclusive societies. Health inequities refer to unfair and avoidable differences in health or healthcare resources caused by poor governance.^6^ The words evacuation, transport, and relocation are used interchangeably in the paper to refer to long-distance move of patients by medical professionals from home to a receiving hospital following established procedures for patient safety and infection control. COVID-19 case and death data were derived from daily reports by Sina.com, which are based on daily reports by the National Health Commission of China and Health Commissions in all China’s 31 provincial level units. The data refer to confirmed cases, those that were tested positive and with symptoms. Fatality refers to percent of deaths among confirmed cases of COVID-19.

## Healthcare System Capacity Overwhelmed: Lockdowns Led to Health Inequities and Worsened the Outbreaks in Wuhan and Hubei

As of April 24, 2020, China had reported 82 804 cases of COVID-19 with 4632 deaths.^7^ Hubei province and its capital city Wuhan bore the majority of the burden (Table). Case fatality in Wuhan and Hubei are several times higher than in the rest of China. Based on the 2018 population, published in 2019 by the Statistical Bureau of China, infection rate in Hubei was over 68 times higher than that in its six neighbors (Human, Jiangxi, Anhui, Henan, Shaanxi, and Chongqing), while the death rate was over 631 times higher. These rates are undoubtedly on the lower end of the estimate, given the number of unconfirmed COVID-19 patients who died outside hospitals and might not have been fully accounted for in the official statistics in Hubei.^8^

**Table:** Total Accumulated Confirmed COVID-19 Cases and Deaths as of April 24, 2020^7^ Versus Medical Resources Before the Outbreak^9,10^ by Location, China

**Location**	**Cases** **No. (%)**	**Deaths** **No. (%)**	**Fatality** **(%)**	**Infection Rate (/100 000)**	**Death Rate (/Million)**	**Medical Worker Cases** ^11^ **No. (%)**	**Beds for Infectious Diseases** **No. (%)**	**All Hospital Beds (1000)** **No. (%)**	**Medical Workers (1000)** **No. (%)**
Wuhan	50 333 (60.79)	3869 (83.53)	7.69	569.55	437.80	2825 (82.80)	1399^a^ (1.06)	78.5 (4.95)	94 (2.38)
Hubei	68 128 (82.28)	4512 (97.41)	6.62	115.14	76.25	3373 (98.86)	4629 (3.52)	95 (6.00)	179 (4.51)
Hubei’s 6 neighbors	5801 (7.01)	42 (0.91)	0.72	1.68	0.12	4 (0.12)	27 806 (21.12)	424 (26.77)	858 (21.65)
Outside Hubei	14 676 (17.72)	120 (2.59)	0.82	1.48	0.12	39 (1.14)	127 026 (96.48)	1489 (94.00)	3786 (95.49)

Abbreviation: COVID-19, coronavirus disease 2019. ^a^These are total beds in the two infectious diseases hospitals in Wuhan.^12^ This could be an underestimate as additional beds might exist in some other hospitals.

The main cause of these shocking geographical disparities in COVID-19 outcomes is that Wuhan and Hubei shouldered the outbreaks with a fraction of the nation’s medical resources (Table). Wuhan had just two infectious diseases hospitals with 1399 beds. Although general hospitals were hastily converted, new hospitals were constructed to counter the patient surges, and materials, equipment, and medical workers were rushed into Wuhan, it was impossible to meet the rising demand.^7,13^ The lockdowns initially caused public panic and resulted in many cross-infections at the crowded, overwhelmed hospitals.^8^ Furthermore, the virus spread quickly at poorly-prepared hospitals, infecting a large number of exhausted healthcare workers.^8^ Of the 3416 COVID-19 cases among medical workers nationwide, who had received donations by April 24, 2020, 83% were in Wuhan and 99% in Hubei (Table). Two weeks after the lockdown on January 23, 2020, there was still a severe shortage of hospital beds for COVID-19 patients.^14^ As the lockdowns prevented patients from seeking care elsewhere, health inequities were created.

Healthcare inequities also occurred between COVID-19 and other patients. There was already a shortage of hospital beds before the outbreak in Wuhan. In the first few weeks of the lockdowns, nearly all medical resources were devoted to COVID-19 control. Patients with other diseases, including children and the elderly, were sent home so that more resources could be directed to fighting the virus.^8,14^ Some seriously ill patients were unable to go back to the hospital for months due to shortage of healthcare for non-COVID-19 treatment.^15^ Thus, the health and well-being of these patients was unfairly endangered. Furthermore, the nationwide lockdowns reinforced xenophobia and locational discrimination as neighbors guarded against neighbors. Anyone crossing the city or provincial borders was automatically a suspected COVID-19 carrier and subjected to extensive quarantine. Hubei residents continue to face discrimination today when they return to their employment in other provinces.

## Patient Relocation Could Have Reduced Health Inequities and Saved Lives

The Hubei lockdowns effectively helped other provinces control the spread of COVID-19, as it has been reported.^1-3^ However, when the healthcare system was incapacitated in Hubei, officials should have begun evacuating patients, particularly those with severe cases. In addition, it would have been faster to move patients out of Hubei, rather than move resources into Hubei and build new hospitals. Healthcare workers from outside Hubei would have worked more efficiently at their home hospitals, where they could be closer to their loved ones, than those in Hubei.

While they were vastly overwhelmed in Hubei, hospitals were in general well below capacity and under-utilized in the rest of China. Outside Hubei, hospital beds tended to be reserved for potential COVID-19 patients during the outbreak and thus were unavailable to most regular patients, except for emergencies. This was probably an overreaction as there were relatively few COVID-19 cases outside Hubei (Table). For example, Hubei’s neighbors Jiangxi had 936 total accumulated cases with one death and Shaanxi had 253 cases with three deaths, as of March 26, 2020.^7^ Outside Hubei, only four provinces had cases over 1000, from 1018 in Hunan to 1448 in Guangdong. Total cases were 640 in Jiangsu with no death, 566 in Beijing, and 468 in Shanghai.^7^ This means that there were many hospital beds left empty outside Hubei, beds that could have accommodated patients from Hubei. In fact, 96% of the total infectious disease hospital beds were in the rest of China, which could have been adequate for accommodating all COVID-19 patients in the country, had they been made available (Table).

It is unknown exactly to what extent patient relocation could have helped save lives, because it was never attempted. However, hypothetical scenarios are proposed here to imitate possible outcomes (Figure). Figure A illustrates what happened in Hubei when the healthcare system was incapacitated, before outside aids came to the rescue afterward. Figure B hypothesizes that patient evacuation would have reduced both cases and deaths markedly when the healthcare system outside Hubei was operating under capacity and would have been able to treat patients transported there. The assumption is that cases and deaths would both decline if hospitals were capable of evaluating and caring for patients, preventing those patients from leaving the hospital and continuing to infect others. It is also assumed that timely patient treatment would reduce cases and deaths, and thus allow the outbreak to be taken under control sooner than it was.

**Figure:**
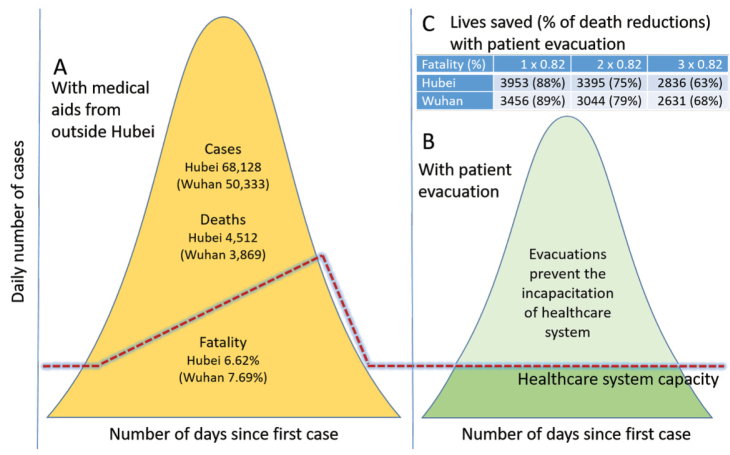
Impact of the Lockdowns on COVID-19 Outcomes in Hubei. (**A**) Hubei with medical aids from outside to boost healthcare capacity but with no patient evacuation. (**B**) A hypothetical scenario of Hubei with patient relocation to “mow” the curve to prevent the incapacitation of the healthcare system. (**C**) The number of lives saved (% of death reductions) with patient transportation, assuming fatalities ranging from one to three times as that outside Hubei but with no total case reductions.

Furthermore, it can be surmised that relocated patients would have faced fatality rates similar to those outside Hubei. Assuming no case reduction but just fatalities ranging from one to three times as that outside Hubei at 0.82%, from 2836 to 3953 lives in Hubei (2097 to 3456 in Wuhan) would have been saved (Figure C). Consequently, deaths would have been reduced by at least 63% and up to even 88% in Hubei (68% to 89% in Wuhan). That is, there was a possibility that nearly 90% of the deaths in Wuhan would have been avoided in the most optimistic scenario. If case reduction were taken into consideration, the outcomes would be even better. Patient relocation would have also helped to ensure healthy lives and promote well-being for non-COVID-19 patients in Hubei as they would have been able to receive essential medical care. It would have been a more sustainable way to flatten the curve.

## Looking Ahead

Debates over the lockdowns in China have focused on their effectiveness in containing the spread of the virus. This paper goes one step further to examine their impacts on fatality and health inequities and proposes patient relocation as an additional way for mitigation. It is unclear why China did not resort to patient evacuation during the crisis, as the highly centralized government had both the authority and transport ability to do so. It is understandable that such a large-scale relocation would have been unprecedented and controversial, due to fear of increased risk of infection in receiving areas. However, COVID-19 patients were securely moved around within Wuhan (eg, inter-hospital transfers). Safe inter-provincial transport should have been feasible. Many countries, including the United States, the United Kingdom, and India, evacuated their nationals from Hubei in February 2020. Later on, China evacuated its nationals from Iran and Britain. Some of the evacuees carried the virus or were suspected to. However, the evacuations were considered safe and there are no reports of increased exposure from evacuees. Furthermore, small-scale evacuations of COVID-19 patients have been conducted in the European Union. For example, French and Italian patients have been moved to Germany and Switzerland for treatment.^16^ There are no known reports of increased infections from these evacuees either. Had it wanted to, China should have been able to safely relocate patients out of Hubei while minimizing additional infections. Yet, the most likely explanation is that China was simply following the conventional infectious disease mitigation playbook that focuses on spatially containing disease transmission.^2,17^ Patient transport, as a way to flatten the curve, would have been more effective than sending medical aids to Wuhan in terms of efficiency in resource allocation and saving lives, though China could have used a combination of both methods. The goal of patient evacuation is to save as many lives as possible by sharing all available healthcare resources. Cities, provinces/states, and even countries may not be adequately prepared for the current COVID-19 or future similar pandemics. The world must form greater alliances and consider unprecedented solutions to manage such emergencies in a more sustainable, inclusive way. As has happened among European countries,^16^ patient relocation may also promote better understanding, friendship, and solidarity among places involved, which, in turn, will contribute to healthy living and well-being for all.

## Acknowledgement

This research was made possible by a nine-month sabbatical leave awarded by University of Central Missouri and a 2019-2020 Fulbright U.S. Scholars Grant (PS00288488) to teach at Northeast Normal University in China. The author also wishes to thank Tiffany Liu for her editing work.

## Ethical issues

Not applicable.

## Competing interests

Author declares that he has no competing interests.

## Author’s contribution

LL is the single author of the paper.
